# Is the Age of Developmental Milestones a Predictor for Future Development in Down Syndrome?

**DOI:** 10.3390/brainsci11050655

**Published:** 2021-05-18

**Authors:** Chiara Locatelli, Sara Onnivello, Francesca Antonaros, Agnese Feliciello, Sonia Filoni, Sara Rossi, Francesca Pulina, Chiara Marcolin, Renzo Vianello, Enrico Toffalini, Giuseppe Ramacieri, Anna Martelli, Giulia Procaccini, Giacomo Sperti, Maria Caracausi, Maria Chiara Pelleri, Lorenza Vitale, Gian Luca Pirazzoli, Pierluigi Strippoli, Guido Cocchi, Allison Piovesan, Silvia Lanfranchi

**Affiliations:** 1 IRCCS, St. Orsola-Malpighi Polyclinic, via Massarenti 11, 40138 Bologna, Italy; chiaralocatelli77@gmail.com (C.L.); agnese.feliciello@studio.unibo.it (A.F.); sonia.filoni23@gmail.com (S.F.); sara.rossi24@studio.unibo.it (S.R.); anna.martelli2@studio.unibo.it (A.M.); giulia.procaccini@gmail.com (G.P.); giacomo.sperti@studio.unibo.it (G.S.); 2 Department of Developmental Psychology and Socialization, University of Padova, via Venezia 8, 35131 Padova, Italy; sara.onnivello@phd.unipd.it (S.O.); francescapulina1@gmail.com (F.P.); marcolinchiara@gmail.com (C.M.); renzo.vianello@unipd.it (R.V.); silvia.lanfranchi@unipd.it (S.L.); 3 Unit of Histology, Embryology and Applied Biology, Department of Experimental, Diagnostic and Specialty Medicine (DIMES), University of Bologna, via Belmeloro 8, 40126 Bologna, Italy; francesca.antonaros2@unibo.it (F.A.); giuseppe.ramacieri@studio.unibo.it (G.R.); maria.caracausi2@unibo.it (M.C.); mariachiara.pelleri2@unibo.it (M.C.P.); lorenza.vitale@unibo.it (L.V.); pierluigi.strippoli@unibo.it (P.S.); 4 Specialisation School in Anesthesia, Intensive Care and Pain Care, University of Modena and Reggio Emilia, Largo del Pozzo, 71, 41125 Modena, Italy; 5 Department of General Psychology, University of Padova, via Venezia 8, 35131 Padova, Italy; enrico.toffalini@unipd.it; 6 Medical Department, Maggiore Hospital, Largo Nigrisoli 2, 40133 Bologna, Italy; gianlucapirazzoli1@gmail.com; 7 Neonatology Unit, St. Orsola-Malpighi Polyclinic, Department of Medical and Surgical Sciences (DIMEC), University of Bologna, via Massarenti 9, 40138 Bologna, Italy; guido.cocchi@unibo.it

**Keywords:** Down Syndrome, developmental milestones, development

## Abstract

Down Syndrome (DS) is the most common genetic alteration responsible for intellectual disability, which refers to deficits in both intellectual and adaptive functioning. According to this, individuals with Down Syndrome (DS) reach developmental milestones (e.g., sitting, walking, and babbling) in the same order as their typically developing peers, but later in life. Since developmental milestones are the first blocks on which development builds, the aims of the current study are to: (i) expand the knowledge of developmental milestone acquisition; and (ii) explore the relationship between developmental milestone acquisition and later development. For this purpose 105 children/adolescents with DS were involved in this study, divided in two groups, Preschoolers (*n* = 39) and School-age participants (*n* = 66). Information on the age of acquisition of *Sitting, Walking, Babbling,* and *Sphincter Control* was collected, together with cognitive, motor, and adaptive functioning. *Sitting* predicted later motor development, but, with age, it became less important in predicting motor development in everyday life. *Babbling* predicted later language development in older children. Finally, *Sphincter Control* emerged as the strongest predictor of motor, cognitive, language, and adaptive skills, with its role being more evident with increasing age. Our data suggest that the age of reaching the milestones considered in the study has an influence on successive development, a role that can be due to common neural substrates, the environment, and the developmental cascade effect.

## 1. Introduction

Down Syndrome (DS) or trisomy 21 [1] is characterized by the presence of an extra full or partial copy of chromosome 21 [2] and is the most common genetic alteration responsible for intellectual disability (ID). According to criteria in the Diagnostic and Statistical Manual of the American Psychiatric Association [3], ID not only refers to intellectual deficits, but also to deficits in adaptive functioning. Further, children with DS show marked motor deficits [4] related to specific syndrome characteristics such as hypotonia [5]. Other more constant and typical features of DS are craniofacial dysmorphisms, together with other variable signs and symptoms such as cardiac malformations and delayed growth [6,7,8,9,10,11]. Considering intellectual functioning, the great majority of individuals with DS show intellectual disability ranging from mild to severe, with a mean Intellectual Quotient (IQ) of 50 and a mental age that rarely exceeds 8 years of age [12]. It is important to note that while mental age continues to increase over time, IQ decreases, probably due to a slower rate of development in individuals with DS compared to their Typically Developing (TD) peers [13]. DS is associated to a relative weakness in speech, language, and verbal abilities as well as particular aspects of memory and to relative strengths in visuo-spatial skills, imitation, and social learning, e.g., [13,14,15].

Similar to cognitive development, difficulties in adaptive skills are visible from early in life and the development of children with DS follows the same trajectory of TD individuals, but at a slower pace [16]. Previous research shows a peak and valleys profile with weaknesses in communication and motor skills, while socialization emerges as a strength [17,18]. Evidence of these developmental profiles can be found early in development [19]. Children with DS generally reach developmental milestones in the same order as their TD peers, but at later chronological ages [20]. The slower rate is due to genetic, neurodevelopmental, and other biomedical influences (e.g., premature birth, congenital heart defects, and sleep dysregulation) and might be influenced by environmental factors, such as intervention experiences or the level of caregiver responsivity [21,22,23]. Considering the language domain, children with DS mainly communicate through gestures, vocalizations, facial expressions, and other movements between 12 and 18 months [24,25]; the onset of canonical babbling (consonant–vowel combinations) is delayed and continues into the second year of life [26] and first words appear, on average, between 18 and 36 months [27]. With regard to motor developmental milestones, several studies have shown that head control is achieved at approximately 6 months, sitting emerges from 8.5 to 15.2 months, crawling from 13.1 to 23.1 and walking from 19.7 to 36.3 [4,28,29]. After the age of 7 months the delay respective to TD peers seems to be less pronounced [20]. As for the other domains, self-care skills (e.g., self-feeding and getting dressed) are acquired later than TD children [30], as well as sphincter control which ranges from 3 years of age up to 4–5 years [31]. In line with this literature, the first aim of our study is to contribute knowledge related to early skill acquisition in individuals with DS, focusing on four developmental milestones: sitting, walking, babbling, and sphincter control.

Research on TD children has demonstrated that early stages of development (and developmental milestones) are related to later development. Early stages of motor development are correlated to later motor development, e.g., [32], but also to later cognitive development and school achievements. For example, Bornstein [33] showed that infants who were more advanced in motor development and who explored more actively at 5 months of age achieved higher levels of cognitive functioning and higher academic levels later in development. Moreover, Murray [34] showed a significant linear relationship between the onset of standing and adult categorization, with better categorization in individuals that started to stand earlier. Along this line Piek [32] demonstrated that gross motor trajectories from birth to 4 years predict later cognitive performance, even when other variables, such as SES, are controlled for. Conversely, Gaysina, Maughan, and Richards [35] showed that delayed gross motor development in infancy (ages at standing and walking) predicted a risk of reading impairment at 11 years of age, even after confounding factors were controlled for. Moreover, early motor milestones, such as sitting and walking are related to language and communication skills. For example, Walle and Campos [36] demonstrated that the acquisition of walking was associated with an increase of language, both receptive and expressive, independent of age. Moreover, Viholainen [37] showed that infants with more advanced motor development during the first year of life had larger vocabularies at 3 and 5 years of age and more advanced reading skills at age 7. In addition, several studies have shown that independent walking increases the frequency of prelinguistic behavior, such as vocalizations, gestures, facial expressions directed toward adults and the initiation of joint engagement, important prerequisites for the development of expressive language [38,39]. Conversely, delayed or absent canonical babbling is related to later speech and language problems, e.g., [40,41].

Focusing on DS, the delays in the onset of developmental milestones are well known, but the relationship between these and later development is not completely clear. Previous studies showed a relationship between motor development and cognitive and language development. For example, Yamauchi [42] showed that in 1 to 3 years old children with DS, motor development is correlated with both cognitive and language development; the strength of this relationship increases with age. Moreover, the achievement of walking has a positive impact on their later cognitive and language development. Supporting this, Fidler [43] showed that a more active exploratory profile in infants with DS is related to higher age equivalent scores in motor, cognitive and communication development. However, the relationship between motor skills and language/cognitive development is not very clear in individuals with DS. In fact, Kim [28] did not find a significant relationship between motor and cognitive/language development in DS while Malak [44] found this relationship only in young children (<3 years of age).

Focusing on language milestones, Lynch [45] showed that the age at onset of babbling for infants with DS was correlated with their scores at 27 months of age for social-communication development. Moreover, Cobo-Lewis [46] demonstrated an association between babbling and motor behaviors, such as hand-banging in infants with DS.

However, at the moment, evidence about the relationship between developmental milestones and later development is not very clear for individuals with DS, also considering that the majority of the studies described above are only correlational, not longitudinal.

For this reason, in the present study we consider four developmental milestones: sitting, walking, babbling, and sphincter control, and we explore their relationship with later motor, cognitive, communication, and adaptive behavior. In particular we have chosen sitting and walking because they are two important milestones of motor development that have previously been determinated to be related to later cognitive and language development in TD individuals [32,33]. We have selected babbling since it has been demonstrated to be a predictor of later speech and language problems [40,41]. Finally, we have chosen to consider sphincter control since it is an important milestone for personal autonomy. We refer to sphincter control as bladder and bowel control and, consequently, not needing to wear diapers at least during daytime. Hence, given the importance of early milestones in development, we believe that understanding their connection with subsequent cognitive, adaptive, and motor development in DS is important in order to predict future outcomes and to better understand what aspects need to be prioritized in early interventions.

In conclusion, the present study, focusing on children and adolescents with DS, aims to:

(i) expand the knowledge on the age of acquisition of developmental milestones; on the basis of previous studies we could expect a mean onset of sitting at 10 months [20,28,29], walking at 27 [28,29] and babbling at 12–16 months [26,27,28,29,30,31,32,33,34,35,36,37,38,39,40,41,42,43,44,45], while the mean age of reaching sphincter control is not very clear (Dolva [31] suggested between 60 and 70 months).

(ii) explore the relationship between babbling, sitting, walking and sphincter control and later motor, cognitive, communication, and adaptive development. This will be investigated separately in preschoolers and school-age participants, considering that in order to use assessment tools appropriate to the chronological age and cognitive level of the child, slightly different tests were used in the two groups. Considering the previous literature on TD individuals and those with DS, we expect not only intra domain relationships (with sitting and walking predicting later motor development, babbling predicting later language, and communication development and sphincter control predicting adaptive behaviors), but also inter domain correlations. In particular, on the basis of the previous literature on TD children, we could expect sitting and walking to also predict later cognitive and language/communication development. However, since the language development trajectory is atypical in DS it is important to verify whether the relationships between different domains of development found in TD children are also the same in those with DS.

## 2. Materials and Methods

### 2.1. Participants

A total of 105 children/adolescents with DS were recruited from February 2014 to July 2019 during their annual follow-up at the Unit of Neonatology of St. Orsola-Malpighi Polyclinic in Bologna, Italy. All of the children and adolescents with DS receive annual follow-up in this unit. The inclusion criteria were a diagnosis of DS with homogeneous or mosaic trisomy 21 and an age ranging between 3 and 17 years. Exclusion criteria were the presence of severe autistic symptoms that interfered with direct assessment of the child. The final sample was composed of 65 males and 40 females. Participants were divided in two groups by age and school attendance: 39 were in the Preschooler group (MCAmonths = 54.9, SDCAmonths = 12.07), 66 in the School-age group (MCAmonths = 135.02, SDCAmonths = 31.19). In the Preschooler group, 92% of participants (*n* = 33) were Caucasian and regarding the parents’ educational level, 95% of mothers (*n* = 37) had a college degree or higher and 87% of fathers (*n* = 34) had a college degree or higher. In the School-age group, 95% of participants (*n* = 63) were Caucasian and regarding the parents’ educational level, 80% of mothers (*n* = 53) had a college degree or higher and 64% of fathers (*n* = 42) had a college degree or higher. All the 39 and 66 participants are available in the OSF repository (https://osf.io/fj3xg/?view_only=b6d878573f0a4e70a2d1ba1c739f8972, accessed on 23 March 2021).

### 2.2. Procedure

The present study was approved by the independent Ethics Committee of the University Hospital St. Orsola-Malpighi Polyclinic, Bologna, Italy (approval no. 39/2013/U/Tess). For all participants involved in the present study, written informed consent was obtained prior to the start of the study. All methods were performed in accordance with the Ethical Principles for Medical Research Involving Human Subjects of the Helsinki Declaration. Clinical data and information on children’s development were collected for each participant.

### 2.3. Measures

#### 2.3.1. Clinical Data

Clinical data were obtained during the routine follow-up visits at the Unit of Neonatology of St. Orsola-Malpighi Polyclinic in Bologna, Italy. A clinician filled in a specific form in which English standard medical terminology was used. Personal, genetic, diagnostic, clinical, and developmental information from both the neonatal period and the time of the visit were collected. Moreover, parents were interviewed regarding the age at which the child started babbling (“Babbling”), sitting without support (“Sitting”), walking without assistance (“Walking”), and controlling sphincter and urination (“Sphincter control”). 

#### 2.3.2. Developmental Measures

From October 2017 to 10 September 2019, a developmental assessment was carried out at the Department of Developmental Psychology and Socialization, University of Padova, Padova, Italy. According to the age group, slightly different tests have been used to directly and indirectly assess developmental outcomes.

### 2.4. Griffiths-III

For children between 3 and 6.11 years of age, a direct measure of general development was considered, the Griffiths-III scales [47], that allow the assessment of 5 developmental domains: Foundations of Learning (A Scale), Language and Communication (B Scale), Eye and Hand Coordination (C Scale), Personal-Social-Emotional aspects (D Scale), and Gross Motor Skills (E Scale). In particular, Foundations of Learning assesses the development of thinking; Language and Communication measures overall language development, including expressive language, receptive language, and communication skills; Eye and Hand Coordination considers fine motor skills, manual dexterity, bimanual coordination, and visual perception skills; Personal-Social-Emotional measures constructs relating to the child’s developing sense of self and growing independence, interactions with others, adaptive behavior and aspects of early emotional development; finally, Gross Motor assesses postural control, balance, and gross body coordination, among other abilities.

Raw scores were converted into Age Equivalent (AE) scores.

A and B Scale values and the IQ of 30 subjects had already been shown in Antonaros [48,49] and, in this study, they are indicated with the same DS patient code.

### 2.5. Wechsler Preschool and Primary Scale of Intelligence (WPPSI-III)

From 7 years of age no direct measures of global development are available. This is because in a clinical setting cognitive assessment is preferred in order to confirm the diagnosis of intellectual disability. To assess the cognitive functioning of participants older than 7 years the WPPSI-III [50] was used, following a procedure already used by Antonaros [48,49] Verbal and Non-Verbal scores and the IQ of 52 subjects had already been shown in Antonaros [48,49] and, in this study, they are indicated with the same DS patient code. Although the WPPSI-III is standardized for children from 2.6 to 7.7 years of age, this test is used here for older participants to avoid floor effect and to use a test more in line with the expected mental age of the sample. Raw scores were transformed into AE scores.

For the purpose of this study, 5 subtests were considered (Block Design, Information, Receptive Vocabulary, Object Construction, and Picture Naming), the only ones that all participants were able to understand and perform. These subtests allow the creation of two indexes: Verbal (Information + Receptive Vocabulary + Picture Naming) and Non-Verbal (Block Design + Object Construction).

### 2.6. Developmental Profile 3 (DP-3)

In addition to direct measures, an indirect measure of development was adopted for all 105 participants: The Development Profile 3 (DP-3) [51]. The DP-3 is a questionnaire that allows the investigation of 5 different developmental areas: Motor, Adaptive Skills, Socio-Emotional, Cognitive and Communication, starting from everyday life behaviors. The questionnaire was completed by parents and raw scores were converted into AE scores.

### 2.7. Vineland Adaptive Behavior Scales-Survey Interview Form-Second Edition (VABS-II)

The VABS-II [52], a semi-structured parent interview, was adopted for all the 105 participants to measure adaptive functioning across 4 domains: Communication, Daily Living Skills, Socialization, and Motor Skills. Individual items are scored on a scale from 0 to 2, which indicates the frequency with which the child shows the skill with independence: usually (2), sometimes (1), or never (0). According to the test characteristics, Motor Skills were assessed only in the Preschooler group. Raw scores were transformed into AE scores.

### 2.8. Statistical Analyses

The clinical and cognitive data were entered into an Excel spreadsheet and analyzed using R [53].

To explore the onset of developmental milestones (Sitting, Babbling, Walking, and Sphincter Control), descriptive statistics were calculated both separately for the two groups and for the whole sample. In addition, correlations between developmental milestones were calculated.

To describe the relationship between milestones and later development separately in the two groups, correlations between developmental milestones and variables describing later motor, cognitive, communication, and adaptive development (Griffiths-III, WPPSI-III, DP-3, and Vineland II scales) were calculated.

To explain variability in development, a series of linear regression models, one for each variable considered (Griffiths-III, WPPSI-III, DP-3, and Vineland-II scales), was implemented for each group. The models are reported in Table 1. These models were calculated considering only the children with all the measures available (30 preschoolers and 53 school-age participants).

The same models were run in the two groups. However, it is important to note that in the Preschooler group the variable Sphincter Control was considered as a dichotomous variable (with 0 = sphincter control not achieved; 1 = sphincter control achieved). This was done because only a limited number of preschoolers had fully acquired this milestone. In the School-age group this variable was considered continuous, similar to all the other milestones. A model selection approach based on Bayesian Information Criterion (BIC) [54] was adopted for these models, using the BIC function of the package “stats”. The BIC was computed for each model and the model with the smallest criterion value was selected. Results were interpreted in terms of BIC and explained variance. We also reported Bayes factors (BF10), expressing the probability of the data given H1 relative to H0 (i.e., values larger than 1 are in favor of H1 whereas values smaller than 1 are in favor of H0) [55]. H1 represents the best model, while H0 the best model without the predictor of interest. Moreover, following a popular rule-of-thumb in the interpretation of the BF, we considered BF between 1 and 3 as indicative of only anecdotal evidence, BF > 3 as indicative of moderate evidence, and any BF > 10 as indicative of strong evidence [56,57].

## 3. Results

### 3.1. The Onset of Developmental Milestones

The mean, standard deviation and median age in months of milestone onset for children with DS are presented in Table 2 for both groups separately and the two groups together. Considering the 39 preschoolers, only 36 reached Sitting, 33 Babbling, 35 Walking, and 16 Sphincter Control. The others had not yet reached these specific milestones at the age of the assessment.

Considering the 66 school-age children/adolescents, information about fundamental development skill achievement is available for 59 (Sitting), 56 (Babbling), 65 (Walking), and 61 (Sphincter Control). This is due to some parents not remembering the age of milestone achievement.

As a whole the mean age of sitting is 9.45 months, of walking 24.37 months, of babbling 14.90 months, and of sphincter control 43.73 months. A certain variability is observed in the age of reaching these developmental milestones, particularly for sphincter control (22.27 months).

Correlations between developmental milestones in the whole sample are reported in Table 3. All milestones resulted intercorrelated. The majority of the correlations were of moderate intensity. A high correlation was present between *Sitting* and *Babbling*.

### 3.2. Developmental Milestones and Later Development

#### 3.2.1. The Preschooler Group

The correlations between developmental milestones and later development in Preschoolers are reported in Table 4. The table reports Pearson coefficients for the correlations between *Sitting*, *Walking*, *Babbling*, *Sphincter Control*, and variables describing later development. Biserial correlations were run for sphincter control since it has been considered as a dichotomous variable, due to the fact that a high number of children in this group did not yet show sphincter control.

Moderate negative correlations emerged between *Sitting* and subscales evaluating motor development assessed in a direct (*Griffiths-III Gross Motor Skills*) and indirect (*VABS-II Motor Skills*) way. *Sitting* resulted also moderately and negatively correlated to *VABS-II Communication*. A moderate correlation emerged between *Babbling* and *VABS-II Communication*, although not significant due to the relatively low number of sample participants. No significant correlations were found in preschoolers between *Walking* and later development. Finally, *Sphincter Control* moderately correlated with *DP-3 Socio-Emotional* and *DP-3 Adaptive Skills*, with children having reached this milestone at higher levels of these two variables related to adaptive function.

To explain variability in development of the Preschooler group, a series of linear regression models was implemented. A model selection approach based on BIC was adopted. For each *Griffiths-III*, *DP-3*, and *VABS-II* scale (corresponding to a specific developmental domain), the best model together with the explained variance (adjR^2^), BF, the standardized coefficients and its confidence intervals (in brackets) are reported in Table 5.

The model containing *Sitting* was the best one to explain *Griffiths-III Gross Motor Skills* (with moderate evidence) and *VABS-II Communication* (with anecdotal evidence). The model containing *Sitting* and *Babbling* was the best predictor of *VABS-II Motor skills*, where the evidence was strong in favor of *Sitting*, anecdotal in favor of *Babbling*. The model containing *Sphincter Control* was the best for *DP-3 Motor*, *Socialization,* and *Adaptive* scales, where the evidence in favor of the predictor was anecdotal for the first two, moderate for the last one. Noteworthy, the standardized ß of *Sphincter Control* was positive because this developmental milestone was a dichotomous variable (0 = sphincter control not achieved; 1 = sphincter control achieved). Therefore, those that have acquired sphincter control demonstrated higher developmental levels in these domains. The null model was the best predictor for all the other variables.

The BIC values for all models are reported in Table S1 of the Supplementary Material. In addition, the correlation between developmental milestones for the Preschooler group are reported in the Supplementary Material, Table S2.

#### 3.2.2. The School-Age Group

The correlations between developmental milestones and later developmental variables are reported in Table 6. The table reports Pearson coefficients for the correlations between *Sitting*, *Walking*, *Babbling*, *Sphincter Control,* and variables describing later development.

*Sitting* showed a close to moderate negative correlation with *WPPSI-III Non-Verbal* and *DP-3 Adaptive Skills*. *Babbling* was moderately and negatively correlated to cognitive and communication variables (*WPPSI-III Verbal* and *Non-Verbal*, *DP-3 Cognitive,* and *Communication*). *Walking* showed moderate and negative correlations to *WPPSI-III Non-Verbal, DP-3 Motor,* and *DP-3 Adaptive Skills*. Finally, *Sphincter Control* moderately and negatively correlated with almost all the subscales except the *DP-3 Socio-Emotional* subscale.

To explain variability in development of the School-age group, a series of linear regression models was implemented. A model selection approach based on BIC was adopted. For each *WPPSI-III* index, *DP-3,* and *VABS-II* scale (corresponding to a specific developmental domain), the best model together with the explained variance (adjR^2^), BF and standardized coefficients are reported in Table 7.

The model containing *Sphincter Control* was the best one for almost all the dependent variables. The evidence in favor of *Sphincter Control* was anecdotal for the *DP-3 Socio-Emotional* scale, moderate for the *WPPSI-III Non-Verbal* index and *VABS-II Socialization*, strong for *DP-3 Motor*, *Adaptive Skills, Cognitive,* and *Communication,* as well as for *VABS-II Communication* and *Daily Living Skills*.

The model containing *Sitting*, *Babbling,* and *Sphincter Control* resulted the best predictor of the *WPPSI-III Verbal* index; the evidence in favor of the predictors was strong. However, given the strong correlation between the predictors in the model (particularly between *Sitting* and *Babbling)*, these estimates should be taken with caution and the particular case of *Sitting* is likely to be a false positive.

The BIC values of all models are reported in Table S3 of the Supplementary Material. In addition, the correlations between developmental milestones for the School-age group are reported in the Supplementary Material, Table S2.

## 4. Discussion

### 4.1. The Onset of Developmental Milestones

The first aim of the present study is to contribute knowledge on the acquisition of developmental milestones in DS. Considering motor development in the study sample, children acquired sitting at approximately 9 months of age and walking at approximately 24 months. These ages are in line or slightly in advance with respect to previous studies. In fact in previous studies the reported age of sitting was 10 months [29] or 11 months [20,21,22,23,24,25,26,27,28] and 26 [28] or 28 months [29] for walking. These small discrepancies could be attributed to a certain degree of interindividual variability that also appears in our sample (6/7 months). Considering language development, the mean age of acquisition of babbling is approximately 15 months, confirming that in children with DS the onset of babbling is delayed and continues into the second year of life [26]. Furthermore, in this case the interindividual variability is quite high (approximately 10 months). Finally, the mean age of sphincter control acquisition is approximately 44 months. This age is precocious with respect to what is reported by Dolva [31] where most of the children between 60 and 70 months were still wearing diapers. It is important to note that for this variable the interindividual variability is very high (22 months) and that 23 children in the younger group had not yet acquired sphincter control.

All these results on developmental milestone acquisition are consistent with the fact that children with DS develop at a slower rate compared with TD children and for this reason milestones are acquired later with respect to their TD peers, e.g., [58]. Moreover, our results are in line with recent research on DS underlying high heterogeneity in development, e.g., [21].

### 4.2. The Role of Developmental Milestones

The second aim of the present study is to determine the relationship between developmental milestones and the subsequent development of the child.

For the preschoolers, sitting resulted to be a significant predictor of later gross motor development, both assessed directly or indirectly through parent reports of everyday life behaviors. Therefore, the earlier a child with DS started to sit, the earlier he/she started to walk, run, and have stronger leg muscles that allow him/her to jump and have better general coordination or balance, all skills assessed in *Griffiths-III Gross-Motor* scale. However, in the School-age group, sitting becomes less important in predicting everyday life motor development as assessed through parents’ reports. In this group walking showed a moderate correlation with later development, although when considered in a model with other variables, it was not a significant predictor.

Moreover, motor milestones resulted to also be related to other developmental domains. In fact in preschoolers the age of onset of sitting showed a moderate correlation and an anecdotal predictive power of later communication skills as described by parents (*VABS-II Communication*). As suggested by Iverson [59], sitting in TD children seems to be related to the onset of babbling. In fact, being seated permits deeper breathing and more consistent subglottal pressure than a supine position. Moreover, keeping the head upright alters the position of the spine and the vocal tract curve and the tongue falls to a more forward position in the oral cavity. This in turn enhances the production of Consonant–Vowel (CV) segments. Apparently this process is also the same for children with DS; in fact in our study a high correlation emerged between sitting and babbling. In addition, in the sitting position, children have their hands free to explore the objects around them and this allows more opportunities to interact with parents, increasing joint attention, an important precursor of communication, both verbal and non-verbal. In addition, as the child can see what is around him/her, there is an increase in language learning opportunities, since the child can direct the attention of parents to objects and parents in turn name them [60].

In the School-age group, motor milestones showed a moderate relation with later cognitive and adaptive development, although when considered in a more complex model these variables did not show a predictive role. On one hand, our results are in line with the results of previous studies conducted on TD children [32,61,62] as well as those with DS [42], showing a relationship between motor milestones and later cognitive development. On the other hand, in the complexity of development, other variables, such as babbling and mainly sphincter control predict subsequent cognitive development in individuals with DS.

Considering language milestones, in preschoolers babbling showed a moderate correlation to later everyday life communication, while in school-age participants it resulted moderately correlated with later communication and cognition. Considered together with other developmental milestones it emerged to be one of the significant predictors of later verbal cognitive development. This result is in line with previous studies on TD children [40,41], as well as on children with DS [45,46] showing a correlation between babbling and later language development that in turns shapes verbal cognitive development, since it is well known that language shapes thinking.

In addition, our data suggest that the onset of sphincter control has an important role in development. Preschoolers that have already acquired sphincter control have better motor, adaptive, and socio-emotional developmental levels. However, the influence of sphincter control on development is bigger in older children. In fact, in school age children sphincter control resulted as significant predictor of later motor, cognitive, communication, adaptive behavior, and motor development. Sphincter control occurs involuntarily in infants and young children until the age of 3 to 5 years, after which it starts to be regulated voluntarily. It is a complex and highly distributed process that involves pathways at many levels of the brain, the spinal cord, and the peripheral nervous system and is mediated by multiple neurotransmitters [63]. In particular, the regions of activity include the primary and secondary sensory/motor cortices, the insula, as well as the association areas of the brain such as the cingulate gyrus, prefrontal cortex, and the parieto-occipital region [64]. The involvement of the motor cortex explains the relationship found between sphincter control and later motor development. On the other hand, the involvement of the prefrontal cortex on sphincter control might help us to understand its relationship with cognitive development. In fact the prefrontal cortex is fundamental for executive functions (i.e., working memory, planning, shifting, and inhibition), which are predictive of children’s cognitive and adaptive functioning, e.g., [65,66]. In this sense a more mature prefrontal cortex allows the child to have more mature regulatory and planning behaviors that have an impact both on sphincter control and on cognitive and interactive behaviors.

Of course, as suggested by Bettinson [67] the development of toilet skills is the first step for independence from parents, the individual’s first social environment. The more a child acquires the abilities to meet her or his own demands, the more that child can be free from the observation of parents and be more independent. Moreover, the earlier a child shows acquisition of such behavior, the more opportunities he/she has to participate autonomously in typical community events and the fewer are his/her needs of practical support in mainstream educational context [68,69].

Considering the important role that sphincter control has shown in our results in predicting later development, there is another potential hypothesis. Between the four predictors that have been considered in our analysis, sphincter control is the one that has the most direct, voluntary and prolonged intervention from parents. We know that sphincter control is a particularly difficult milestone for children with DS [31]. For this reason, to help the child reach toilet autonomy, parents have to train him/her, often for a prolonged period of time, teaching how to detect signals from the body, how to control bladder and bowel and how to plan a sequence of actions to use the toilet correctly. In this sense sphincter control could be considered a variable more influenced by parents’ attitude toward the education of the child. A potential hypothesis is that parents that decide to face the prolonged effort to toilet train their child earlier are parents that are more prone to stimulate and somehow expose him/her to other developmental domains. Of course, these are only speculations that will be interesting to explore in future studies.

This study is not without limitations. We are aware that the number of participants in our groups (Preschooler and School-age) was small. Moreover since the enrollment in this project was voluntary, it is possible that our participants are a selected sample with particular characteristics, such as with parents highly involved in the education of their children. For these reasons a larger sample size in a future work would be recommended. Another area of weakness of our work is linked to the fact that the age of the onset of milestones has been reconstructed through parents’ report at the moment of the assessment, several years later. It is possible that parents’ reports are approximate; of course, a longitudinal study would allow a more precise assessment of milestone onset. In addition, in our study we only considered four milestones. We believe that it would be important in the future to also explore the role of other motor, language, cognitive, and personal milestones on future development. We believe that this is a very important aspect to explore because not only does it better allow us to understand the developmental profiles of children with DS, but this knowledge of early predictors of later development is very helpful to plan early phenotype-informed interventions that have the potential to influence positive trajectories in community living and participation across an individual’s lifespan. A final limit of our study might be linked to the fact that in our study a part of the test used involves indirect assessment, that is reconstructing development and adaptive behavior through parents’ reports [70]. In this case parents’ affirmations could be influenced by motivational factors, such as social desirability. However, these aspects have been partially accounted for by combining direct and indirect assessment tools that offer the possibility to look at child development from different points of view.

## Figures and Tables

**Table 1:** Models.

	Variables
Model 0	Null model
Model 1	Sitting
Model 2	Walking
Model 3	Babbling
Model 4	Sphincter Control
Model 5	Sitting + Walking
Model 6	Sitting + Babbling
Model 7	Sitting + Sphincter Control
Model 8	Babbling + Walking
Model 9	Babbling + Sphincter Control
Model 10	Walking + Sphincter Control
Model 11	Sitting + Babbling + Walking
Model 12	Sitting + Babbling + Sphincter Control
Model 13	Sitting + Walking + Sphincter Control
Model 14	Babbling + Walking + Sphincter Control
Model 15	Sitting + Babbling + Walking + Sphincter Control

**Table 2:** Descriptive statistics of age of acquisition (in months) of developmental milestones.

	Preschoolers (*n* = 39) M (SD)	School-Age Group (*n* = 66) M (SD)	Total Group (*n* = 105) M (SD)
Sitting	8.44 (2.98) *n* = 36	10.07 (7.28) *n* = 59	9.45 (6.03) *n* = 95
Babbling	12.88 (5.27) *n* = 33	16.09 (11.30) *n* = 56	14.90 (9.61) *n* = 89
Walking	23.59 (5.04) *n* = 35	24.78 (8.96) *n* = 65	24.37 (7.81) *n* = 100
Sphincter Control	33.45 (11.02) *n* = 16	46.43 (23.71) *n* = 61	43.73 (22.27) *n* = 77

**Table 3:** Correlations between developmental milestones.

	Sitting *n* = 95	Babbling *n* = 89	Walking *n* = 100	Sphincter Control *n* = 77
Sitting	-	0.734	0.610	0.534
Babbling	0.734	-	0.442	0.463
Walking	0.610	0.442	-	0.412
Sphincter Control	0.534	0.463	0.412	-

**Table 4:** Correlations between developmental milestones and cognitive, language, adaptive, and motor skills in the Preschooler group. *N* = 30.

	Sitting	Babbling	Walking	Sphincter Control
Griffiths-III Foundations of Learning	−0.080	0.158	−0.011	0.209
Griffiths-III Language and Communication	−0.238	−0.002	0.135	0.307
Griffiths-III Eye and Hand Coordination (*n* = 25. For these variables the number is reduced because 5 participants did not complete the Griffiths-III assessment)	−0.137	−0.040	−0.148	0.280
Griffiths-III Personal-Social-Emotional (*n* = 25. For these variables the number is reduced because 5 participants did not complete the Griffiths-III assessment)	−0.167	0.099	0.105	0.321
Griffiths-III Gross Motor Skills (*n* = 25. For these variables the number is reduced because 5 participants did not complete the Griffiths-III assessment)	−0.445	0.033	0.022	0.266
DP-3 Motor	−0.233	−0.062	0.131	0.331
DP-3 Socio-Emotional	0.167	0.074	0.253	0.400
DP-3 Adaptive Skills	0.093	−0.053	0.222	0.430
DP-3 Cognitive	−0.114	0.063	0.238	0.246
DP-3 Communication	−0.220	−0.300	0.203	0.291
VABS-II Communication	−0.360	−0.161	0.067	0.230
VABS-II Daily Living Skills	−0.239	0.136	−0.07	0.120
VABS-II Socialization	−0.152	0.024	0.097	0.213
VABS-II Motor Skills	−0.397	0.127	−0.098	0.102

**Table 5:** Best model selection in the Preschooler group. *N* = 30.

	Best Model	BF_10_	adjR^2^	Standardized Coefficients
Griffiths-III Foundations of Learning	Null model			
Griffiths-III Language and Communication	Null model			
Griffiths-III Eye and Hand Coordination (*n* = 25 For these variables the numerosity is reduced because 5 participants did not complete the Griffiths-III assessment)	Null model			
Griffiths-III Personal-Social-Emotional (*n* = 25. For these variables the number is reduced because 5 participants did not complete the Griffiths-III assessment)	Null model			
Griffiths-III Gross Motor Skills (*n* = 25. For these variables the number is reduced because 5 participants did not complete the Griffiths-III assessment)	Sitting	BF_10_ = 3.19	16%	Sitting ß = −0.45, (−0.83, −0.59)
DP-3 Motor	Sphincter Control	BF_10_ = 1.04	8%	Sphincter control ß = 0.33, (−0.03, 0.67)
DP-3 Socio-Emotional	Sphincter Control	BF_10_ = 2.53	13%	Sphincter control ß = 0.40, (0.05, 0.76)
DP-3 Adaptive Skills	Sphincter Control	BF_10_ = 4.44	16%	Sphincter control ß = 0.44, (0.09, 0.79)
DP-3 Cognitive	Null model			
DP-3 Communication	Null model			
VABS-II Communication	Sitting	BF_10_ = 1.48	10%	Sitting ß = −0.36, (−0.72, 0)
VABS-II Daily Living skills	Null model			
VABS-II Socialization	Null model			
VABS-II Motor Skills	Sitting + Babbling	Sitting BF_10_ = 12.18 Babbling BF_10_ = 1.21	20%	Sitting ß = −0.54, (−0.91, −0.17) Babbling ß = 0.36, (−0.03, 0.72)

**Table 6:** Correlations between developmental milestones and cognitive, language, adaptive, and motor skills in the School-age group. *N* = 53.

	Sitting	Babbling	Walking	Sphincter Control
WPPSI-III Verbal	−0.273	−0.539	−0.214	−0.492
WPPSI-III Non-Verbal	−0.299	−0.301	−0.274	−0.352
DP-3 Motor	−0.229	−0.107	−0.340	−0.399
DP-3 Socio-Emotional	−0.101	−0.125	−0.112	−0.292
DP-3 Adaptive Skills	−0.294	−0.217	−0.330	−0.541
DP-3 Cognition	−0.249	−0.340	−0.223	−0.575
DP-3 Communication	−0.194	−0.332	−0.213	−0.521
VABS-II Communication	−0.160	−0.260	−0.222	−0.451
VABS-II Daily Living Skills	−0.145	−0.063	−0.185	−0.437
VABS-II Socialization	−0.064	−0.002	−0.149	−0.355

**Table 7:** Best model selection in the School-age group. *N* = 53.

	Best Model	BF_10_	adjR^2^	Standardized Coefficients
WPPSI-III Verbal	Sitting + Babbling + Sphincter Control	Sitting BF_10_ = 23.33 Babbling BF_10_ = 2892.857 Sphincter Control BF_10_ = 90.01	45%	Sitting ß = 0.55, (0.21, 0.89) Babbling ß = −0.76, (−1.00,−0.43) Sphincter ß = −0.45, (−0.70, −0.21)
WPPSI-III Non Verbal	Sphincter Control	BF_10_ = 4.53	11%	Sphincter ß = −0.35 (−0.61,−0.09)
DP-3 Motor	Sphincter Control	BF_10_ = 13.60	14%	Sphincter ß = −0.39, (−0.66,−0.14)
DP-3 Socio-Emotional	Sphincter Control	BF_10_ = 1.46	28%	Sphincter ß = −0.29, (−0.56,−0.02)
DP-3 Adaptive Skills	Sphincter Control	BF_10_ = 1339.431	7%	Sphincter ß = −0.54, (−0.78, −0.20)
DP-3 Cognitive	Sphincter Control	BF_10_ = 5825.499	32%	Sphincter ß = −0.57, (−0.81, −0.35)
DP-3 Communication	Sphincter Control	BF_10_ = 607.8937	26%	Sphincter ß = −0.52, (−0.76, −0.28)
VABS-II Communication	Sphincter Control	BF_10_ = 56.83	19%	Sphincter ß = −0.46, (−0.70, −0.20)
VABS-II Daily Living skills	Sphincter Control	BF_10_ = 37.71	18%	Sphincter ß = −0.44, (−0.70, −0.18)
VABS-II Socialization	Sphincter Control	BF_10_ = 4.95	11%	Sphincter ß = −0.36, (−0.62, −0.09)

## Data Availability

The data that support the findings of this study are available in the OFS repository. https://osf.io/fj3xg/?view_only=b6d878573f0a4e70a2d1ba1c739f8972 (accessed on 23 March 2021).

## Supplementary Materials

The following are available online at https://www.mdpi.com/article/10.3390/brainsci11050655/s1, Table S1: BIC values Preschooler group; Table S2: Correlations between developmental milestones in the Preschooler and School-age group; Table S3: BIC values School-age group. The Supplementary Materials are in the attached file.
