# Supplementary material for: Is the Age of Developmental Milestones a Predictor for Future Development in Down Syndrome?
Source: Brain Sci. 2021 May 18;11(5):655. doi: 10.3390/brainsci11050655 (PMC8157296; doi:10.3390/brainsci11050655)
Supplement: Supplementary file 1 [file brainsci-11-00655-s001.zip › brainsci-1179569-supplementary.pdf]

Supplementary material

Table S1 BIC values Preschooler group

Model	Griffiths A	Griffiths B	Griffiths C (n=25)	Griffiths D (n=25)	Griffiths E (n=25)	DP3 Motor	DP3 Social	DP3 Adaptive	DP3 Cognitive	DP3 Comunication	VABS Comunication	VABS Daily	VABS Social	VABS Motor
0	**183.8367**	**214.0786**	**149.4189**	**182.3136**	147.2321	218.8308	232.7863	219.5171	**219.2041**	**237.8046**	236.4872	**228.3330**	**232.7600**	200.0452
1	187.0475	215.7291	152.1630	184.8227	**144.9197**	220.5530	235.3398	222.6596	222.2147	239.7109	**235.7161**	229.9747	235.4562	198.2993
2	187.2344	216.9223	152.0860	185.2522	150.4390	221.7162	234.2102	221.4057	220.8504	239.9468	239.7521	231.6058	235.8802	203.1591
3	186.4767	217.4797	152.5984	185.2866	150.4244	222.1167	236.0210	222.8346	222.4863	238.3774	239.0993	231.1762	236.1437	202.9561
4	185.8909	214.5057	150.5897	182.8096	148.6207	**18.7499**	**230.9375**	**216.5412**	220.7383	238.5497	238.2601	229.1011	234.7745	203.1335
5	190.4417	218.6466	154.7831	187.7826	148.1370	223.5094	236.5426	224.4573	223.9264	241.9399	239.0390	233.1789	238.6167	201.1870
6	189.1047	218.7832	155.3704	187.1072	146.1531	223.9130	238.7394	225.7627	225.1786	241.3929	239.1085	231.2357	238.5836	**197.9131**
7	189.2154	216.6170	153.5609	185.6272	146.8614	220.9509	232.4207	218.9965	223.9414	240.8721	237.9914	231.1848	237.6996	201.6307
8	189.8364	220.3063	155.3021	188.3109	153.6371	224.9012	237.5738	224.5528	224.2317	239.5690	242.2247	234.3380	239.2791	205.9177
9	188.3826	217.9006	153.7244	185.8458	151.8313	222.0814	234.0177	219.9068	223.9579	239.1475	240.9419	231.7517	238.1377	205.9970
10	189.1938	217.7298	152.7444	185.9469	151.8271	222.0185	233.2560	219.2778	222.9492	241.2537	241.6491	231.9531	238.0865	206.0827
11	192.3987	221.8340	157.9114	190.2389	149.2775	226.9054	239.8724	227.1554	227.1491	242.7718	242.4167	234.0326	241.8286	199.8713
12	191.2435	219.6614	156.7736	188.2072	148.4066	224.3142	235.8216	221.9820	226.8988	242.3702	241.3812	232.3278	240.8283	201.2466
13	192.5136	219.8572	155.6956	188.7662	150.0597	224.2338	234.5864	221.6693	226.1685	243.5943	241.3880	233.9662	241.0203	204.4098
14	191.5388	221.1310	155.9539	189.0214	155.0328	225.3083	236.4987	222.5636	226.2904	241.1016	244.2725	234.3060	241.4666	208.7224
15	194.3061	222.9797	158.8905	191.4134	151.4240	227.6187	237.9410	224.3535	229.3529	244.4100	244.7740	234.3828	244.1961	202.9925

Table S2 Correlations between developmental milestones in the Preschooler and School-age group

	Preschoolers (n=30)	Schoolers (n=53)
Sitting - Walking	−0.055	0.709
Sitting - Babbling	0.407	0.763
Walking - Babbling	0.161	0.496
Sitting - Sphincter Control	-	0.547
Walking - Sphincter Control	-	0.450
Babbling - Sphincter Control	-	0.486

Table S3 BIC values School-age group

Model	WPPSI Verbal	WPPSI Non Verbal	DP3 Motor	DP3 Social	DP3 Adaptive	DP3 Cognitive	DP3 Comunic ation	VABS Comunic ation	VABS Daily	VABS Social
0	462.6839	427.0585	473.7973	494.8722	490.0516	480.5409	491.2665	468.6485	448.7541	455.3266
1	462.5352	426.0744	474.9016	498.3009	489.2435	481.1311	493.1932	471.2392	451.5976	459.0779
2	464.1713	426.9007	471.2445	498.1745	487.9068	481.8160	492.7652	469.9373	450.8707	458.1049
3	448.4967	425.9804	477.1562	498.0039	491.4667	478.0030	489.0327	468.8832	452.5134	459.2967
4	451.9535	**424.0357**	**468.5921**	**494.1086**	**475.6470**	**463.2027**	**478.4581**	**460.5672**	**441.5026**	**452.1373**
5	466.4593	429.5943	475.1978	502.0950	491.4513	484.8561	496.5274	473.9068	454.8207	461.8868
6	448.9690	429.2878	478.2486	501.9707	493.2067	481.9563	492.5010	472.6480	455.2727	462.7663
7	455.9224	427.0245	472.5516	497.7863	479.6163	466.6664	481.5609	463.8156	444.6332	454.6084
8	452.1889	428.7417	474.9132	501.7967	491.6520	481.7392	492.8149	472.1997	454.7805	461.7030
9	446.2818	426.4286	472.1465	498.0760	479.5501	466.5185	481.5477	464.2576	443.9856	454.1737
10	455.8658	427.1605	470.7591	498.0101	479.1728	466.8369	482.2788	464.5367	445.4112	456.0635
11	452.4266	432.6682	478.7791	505.7119	495.4192	485.4434	495.3407	474.8157	458.5773	465.6722
12	**439.9790**	430.3685	475.5315	501.5592	483.4782	467.2752	480.4580	465.3189	447.9447	458.0065
13	459.7829	430.8313	473.9160	501.7498	482.8423	470.6014	485.4817	467.3146	448.4543	458.1261
14	447.9042	430.1338	473.2347	501.9787	482.8596	469.6154	484.9151	468.1988	447.9173	458.0517
15	443.9265	434.0900	477.1796	505.5118	486.7567	471.2453	484.1835	468.5016	451.8254	461.6228
